# The 7BM beamline at the APS: a facility for time-resolved fluid dynamics measurements

**DOI:** 10.1107/S0909049512016883

**Published:** 2012-05-11

**Authors:** Alan Kastengren, Christopher F. Powell, Dohn Arms, Eric M. Dufresne, Harold Gibson, Jin Wang

**Affiliations:** a Center for Transportation Research, Argonne National Laboratory, 9700 South Cass Avenue, Argonne, IL 60439, USA; b Advanced Photon Source, Argonne National Laboratory, 9700 South Cass Avenue, Argonne, IL 60439, USA

**Keywords:** time-resolved, radiography, multilayer monochromator, Kirkpatrick–Baez mirrors

## Abstract

In recent years, X-ray radiography has been used to probe the internal structure of dense sprays with microsecond time resolution and a spatial resolution of 15 µm even in high-pressure environments. Recently, the 7BM beamline at the Advanced Photon Source (APS) has been commissioned to focus on the needs of X-ray spray radiography measurements. The spatial resolution and X-ray intensity at this beamline represent a significant improvement over previous time-resolved X-ray radiography measurements at the APS.

## Abstract 2

The 7BM beamline, a facility for time-resolved fluid dynamics measurements at the Advanced Photon Source, is described.

## 1. Introduction

Spray systems have important applications in internal combustion engines, rocket combustors, paint application and other industries. Optical techniques are commonly used to study the spray structure and droplet sizing. For dilute sprays these optical techniques can be applied effectively. For dense sprays, such as those seen in fuel sprays for diesel engines, the strong scattering of light by the spray droplets makes the use of optical techniques problematic (Sick & Stojkovic, 2001 ▶). While some advanced optical techniques have been applied to such flowfields (Chaves *et al.*, 2004 ▶; Leick *et al.*, 2004 ▶; Schmidt *et al.*, 2009 ▶), these techniques are still under development.

An alternative to optical techniques is X-ray radiography and imaging of spray systems. X-rays are strongly absorbed but only weakly scattered by liquid–gas phase boundaries, providing clear images and quantitative data regarding multiphase flow structure. X-ray techniques have been used to study several fluid flowfields, including planar jets (Kuo *et al.*, 1990 ▶), orifice flow (Birk *et al.*, 1993 ▶), water sprays (Meyer *et al.*, 2008 ▶), cavitation in a model spray nozzle (Giannadakis *et al.*, 2008 ▶) and fuel sprays (Kastengren *et al.*, 2009*a* ▶,*b* ▶). A recent review article describes a long history of using X-ray techniques to probe fluid flowfields (Heindel, 2011 ▶).

A significant limitation to previous X-ray radiography measurements of fluid dynamics has been access to an X-ray source of sufficient intensity to produce time-resolved measurements of spray structure. A synchrotron X-ray source has been used for such measurements in the past (Kastengren *et al.*, 2009*a* ▶,*b* ▶), but with limited time available for measurements. Moreover, the beamline used in previous experiments uses a crystal monochromator with a relatively narrow energy bandwidth. This narrow bandwidth limits the flux available for the current radiography measurements.

A new synchrotron X-ray beamline, the 7BM beamline, has recently been commissioned at the Advanced Photon Source (APS) at Argonne National Laboratory largely dedicated to X-ray radiography of spray systems. This paper will discuss the properties and potential of this beamline in detail.

## 2. Beamline overview

The X-ray source for the beamline is an APS bending magnet. The beamline consists of two end-stations. The 7BM-A end-station houses the front-end optics. A pair of water-cooled 250 µm-thick beryllium windows (aperture 8.8 mm × 145 mm, V × H) are fitted to the entrance of the hutch to isolate the beamline from the storage ring vacuum chamber. This enclosure also houses a set of water-cooled white-beam slits, a monochromator and an optional 0.5 m-long palladium-coated flat mirror (2 µrad slope error, 2.2 Å surface roughness) that can be used at 7 mrad grazing angle to reject high-energy harmonics if necessary. A 400 µm-thick beryllium window terminates the UHV beamline vacuum chamber in the 7BM-B experimental end-station. This window consists of two apertures, one for the direct monochromatic beam and one for monochromatic beam reflected from the flat mirror.

**Figure 1:**
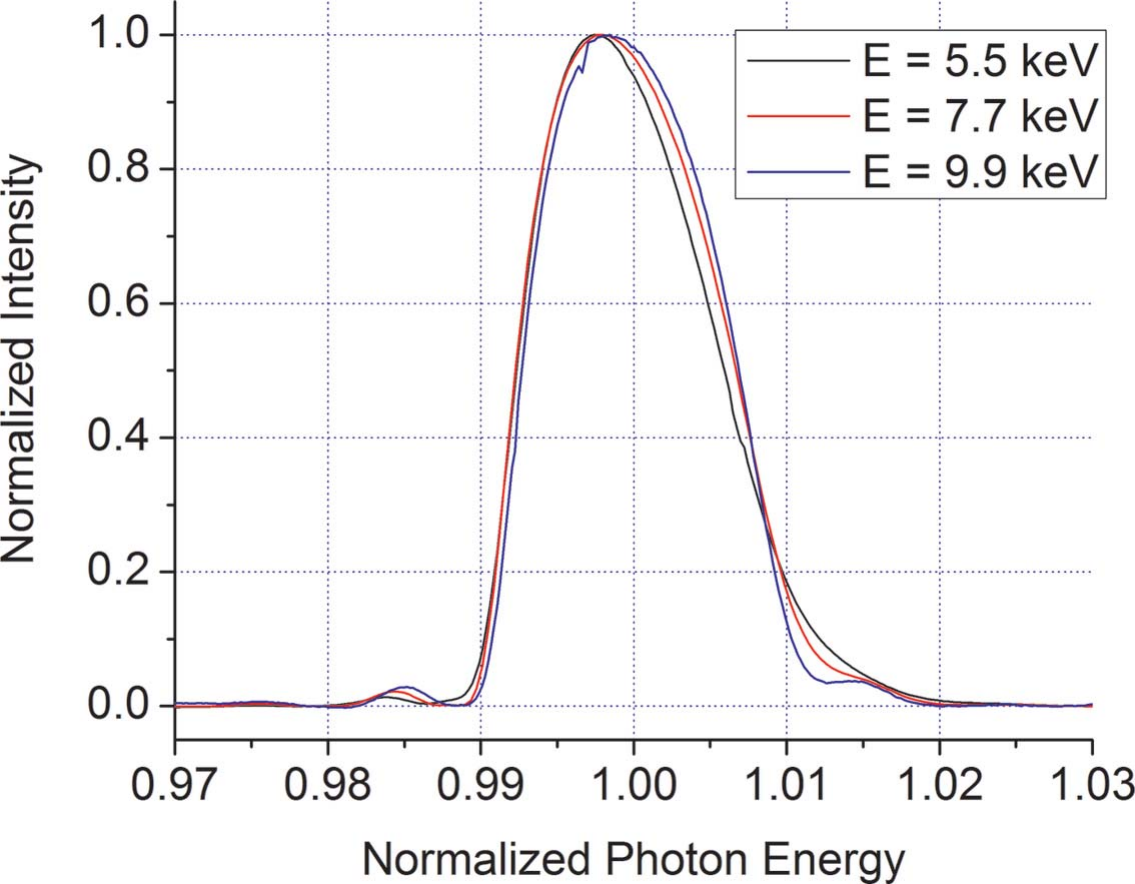
Energy spectrum of the monochromatic X-ray beam for three different average photon energy values. The energy has been normalized by the average energy and the intensity by the peak intensity.

The monochromator used in these experiments is a water-cooled double-multilayer design described previously (Wang *et al.*, 2007 ▶). It is designed with a fixed vertical offset of 35 mm and can accept 3.5 mrad of the bending-magnet radiation horizontally. The current design of the monochromator uses two flat multilayer crystals (*i.e.* without sagittal focusing) with W/B_4_C multilayers, which provide high reflectivity in the energy range desired for spray radiography measurements and a wide energy bandpass. The spectrum of the monochromatic beam is shown in Fig. 1 ▶. The FWHM bandpass is 1.4% and is almost constant as the monochromator energy is changed. This bandpass is well suited to time-resolved radiography experiments: it is wide enough to provide high flux but narrow enough to be treated as virtually monochromatic. The multilayer coatings can also potentially act as a reflection mirror for low-energy (<2 keV) X-rays; owing to the absorption of such X-rays in the beamline beryllium windows and air gaps in the experimental hutch, very little flux is expected at these energies.

**Figure 2:**
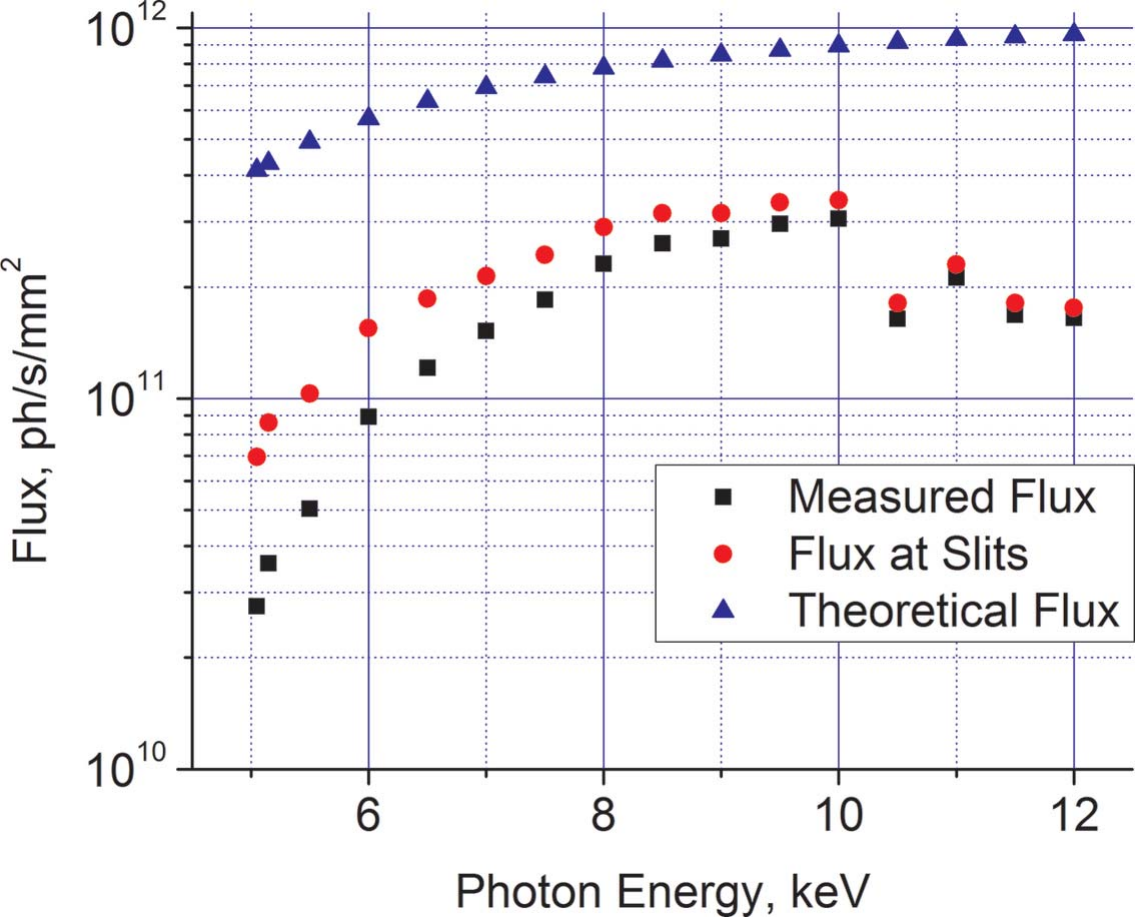
Beamline flux as a function of photon energy. Measurements were performed 36 m from the X-ray source.

The energy range of the monochromator is 5.1–12 keV (wavelength range 1.0–2.4 Å). Fig. 2 ▶ shows the measured flux from the beamline 36 m from the X-ray source at a range of X-ray energies. After accounting for the reflectivity of the multilayers, the beamline flux is a large fraction of the expected flux. The flux generally improves at higher energies, owing to the increased brilliance of the X-ray source and reduced absorption in the beamline windows. This trend does not hold above 10 keV; the tungsten *L*-edges are between 10.2 and 12.1 keV, which reduces the multilayer reflectivity in this region.

**Figure 3:**
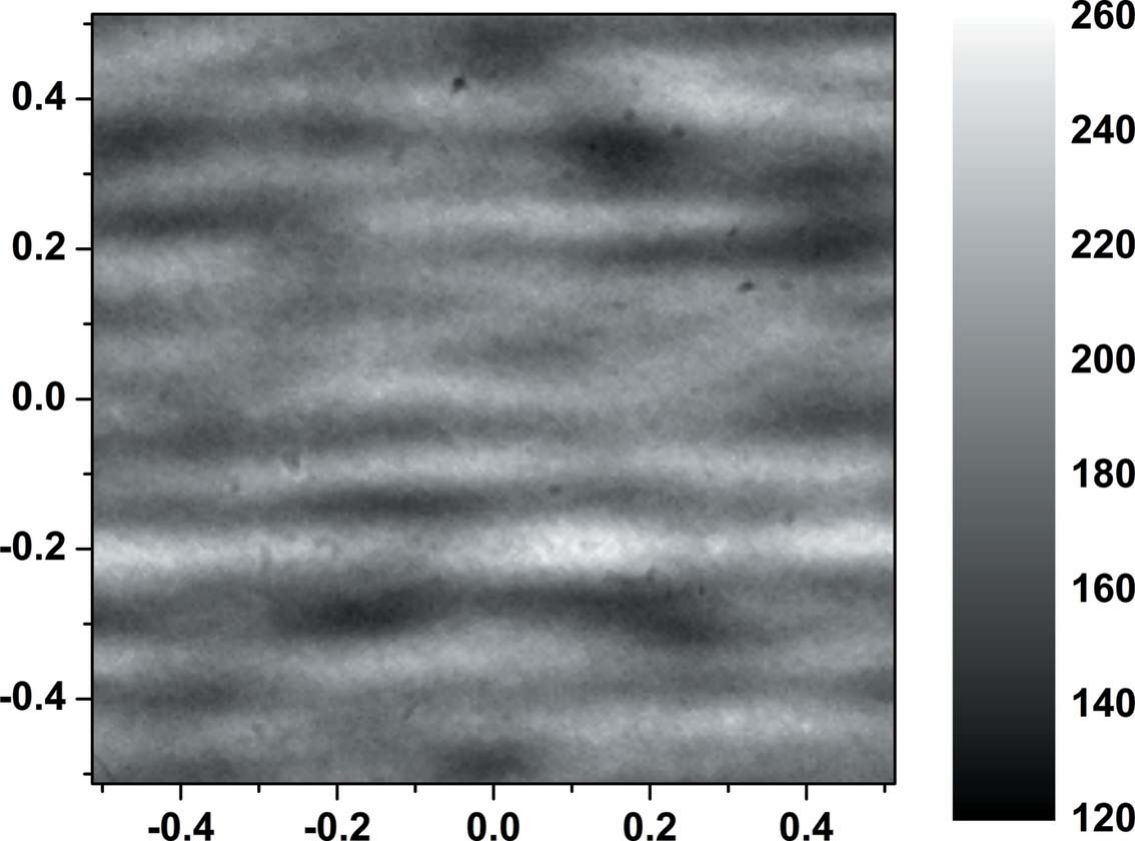
Unfocused beam profile at 10 keV. Image size, 1.03 mm × 1.03 mm; pixel size, 5.2 µm; exposure time, 0.011 s. The intensity scale is in arbitrary units.

The unfocused monochromatic beam contains significant structure owing to imperfections in the multilayer substrates. Fig. 3 ▶ shows an image of a small portion of the unfocused beam captured with a LYSO:Ce crystal and a visible-light microscope at 10 keV. The beam contains a strong horizontal striping pattern, which has been seen in other beamlines employing multilayer monochromators (Rack *et al.*, 2010 ▶). The standard deviation of the intensity is approximately 10% of the mean intensity. This striping, combined with beam motion, interferes with performing the background corrections needed for full-field imaging, which limits the ability of the beamline to perform full-field imaging.

**Figure 4:**
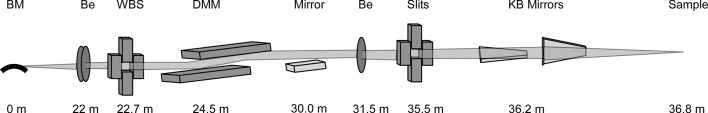
Beamline schematic. BM: bending magnet; Be: beryllium window; WBS: white-beam slits; DMM: double multilayer monochromator. Distances of components from the X-ray source are given below the schematic.

Beamline focusing is achieved with a pair of Kirkpatrick–Baez mirrors procured from IDT. The mirrors are 300 mm long (260 mm optical length) and coated with rhodium. The measured reflectivity of each mirror is greater than 80% from 7 to 10 keV at 5 mrad grazing angle. The focus FWHM of the mirrors is 5 µm × 6 µm, V × H. This is somewhat greater than the expected focus spot size, potentially due to aberrations from the multilayer substrates or limitations in the imaging system used to measure the focus spot size. A schematic of the beamline layout is given in Fig. 4 ▶. Table 1 ▶ gives the pertinent beamline parameters.

## 3. Ancillary equipment

The beamline controls and data acquisition are performed through an EPICS interface. A sample preparation area is available. The beamline is equipped with detectors for high-speed raster-scan radiography, including suitable electronics and software to automate the capture of high-speed radiography data, and an imaging system for low-speed full-field imaging. The beamline is also equipped with a high-capacity chemical exhaust system to aid in performing spray experiments.

## 4. Facility access

The beamline has been built as a partnership between the US DOE Office of Science and the DOE Office of Energy Efficiency and Renewable Energy. A fraction of the available time at the beamline will be available to general users through the APS General User Proposal System. An additional portion of the available time will be devoted to research with applications to transportation and vehicle technologies.

## 5. Highlights

To demonstrate the performance of the beamline, time-resolved radiography has been performed on diesel sprays. The injector is a common-rail injector fitted with a three-hole nozzle, each hole of which is nominally 145 µm in diameter. The injector sprays into a chamber filled with nitrogen gas at room temperature and 5 bar absolute pressure (5.6 kg m^−3^ density). The fuel used is a diesel calibration fluid (Viscor 1487); unlike most previous X-ray radiography measurements, no contrast agents were used for these measurements. Data are obtained point-by-point, with the data at each point representing the ensemble average of 32 to 128 spray events. Approximately 2500 individual spatial locations are measured using a variable-spacing measurement grid. The grid spacing varies from 5 to 140 µm across the spray axis and 0.1 to 2 mm along the beam axis. Owing to the absorption of the spray chamber windows and fill gas, the flux at the X-ray detector was 4 × 10^10^ photons s^−1^ at 8 keV.

**Figure 5:**
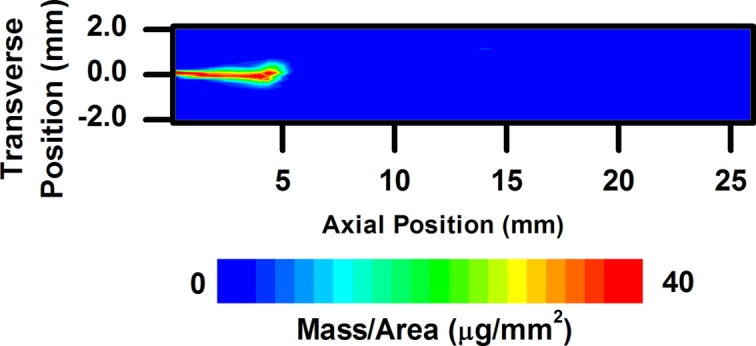
Two-dimensional fuel distribution for a diesel spray 44 µs after the apparent start of injection.

**Figure 6:**
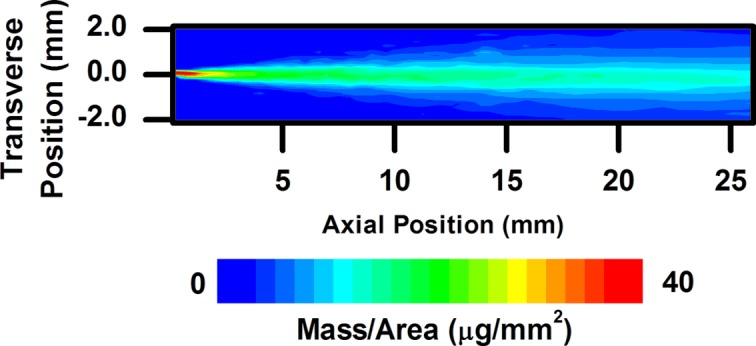
Two-dimensional fuel distribution for a diesel spray 523 µs after the apparent start of injection, during the main portion of the injection event.

**Figure 7:**
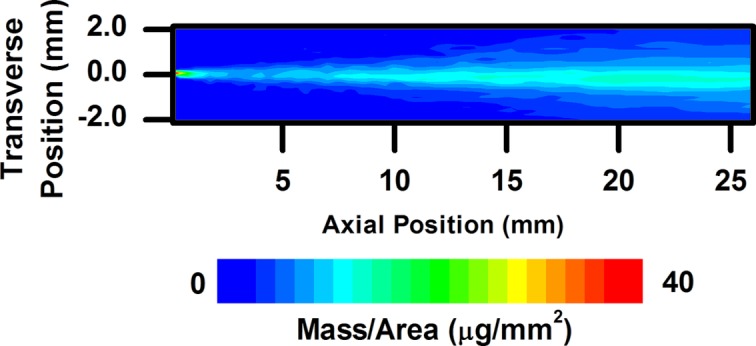
Two-dimensional fuel distribution for a diesel spray near the end of the injection event, 1001 µs after the apparent start of injection.

Fig. 5 ▶ shows the projected density (mass per unit area) of a diesel spray 44 µs after the apparent start of injection (SOI). As has been seen in previous spray radiography measurements, a concentration of fuel exists at the leading edge of the spray. Fig. 6 ▶ shows the spray mass distribution 523 µs after the apparent SOI, when the spray exhibits quasi-steady-state behavior. The mass distribution at the end of injection is displayed in Fig. 7 ▶. It should be noted that the higher flux (by a factor of roughly 20) and spatial resolution (by a factor of three vertically and 25 horizontally) available at the 7BM beamline allow for much more precise measurements of sprays compared with previous measurements, especially in the absence of contrast agent.

## 6. Discussion and conclusions

X-ray radiography of spray systems can provide time-resolved quantitative measurements of regions that cannot be effectively probed using optical techniques. In the past, access to suitable X-ray sources has been problematic. The 7BM beamline at the Advanced Photon Source has recently been constructed to help fill this gap. The beamline provides a tunable (5.1–12 keV photon energy) and monochromatic (1.4% bandpass) beam for easy conversion of X-ray intensity to flowfield density. The beamline can provide a wide uniform beam for imaging experiments. Alternatively, the beam can be focused to a spot size of less than 10 µm × 10 µm for point-by-point measurements. Measurements of diesel sprays performed at the beamline showed significantly better quality than previous measurements at another beamline. This facility will soon be made available to outside researchers, providing a unique resource for the fluid mechanics community.

## Figures and Tables

**Table 1:** Beamline parameters

	
Mirrors	Kirkpatrick–Baez geometry, each 300 mm-long, Rh-coated Si
Monochromator	Double multilayer, W/B_4_C multilayers, 23.75 Å/bilayer
Energy range	5.1–12 keV, Δ*E*/*E* = 1.4% FWHM
Beam size focused	5 µm × 6 µm FWHM at 8 keV
Flux	1.6 × 10^11^ photons s^−1^ at 10 keV, beam focused with mirrors
